# Effects of salinity on pre- and post-fertilization developmental events in the clam *Anomalocardia flexuosa* (Linnaeus, 1767)

**DOI:** 10.1590/1984-3143-AR2023-0005

**Published:** 2023-05-26

**Authors:** Rodolf Gabriel Prazeres Silva Lopes, Ana Paula Rego, Sabrina Melo de Jesus Gomes, Thayane Ramos, Ícaro Gomes Antonio, Maria Raquel Moura Coimbra

**Affiliations:** 1 Departamento de Pesca e Aquicultura, Universidade Federal Rural de Pernambuco, Recife, PE, Brasil; 2 Departamento de Engenharia de Pesca, Universidade Estadual do Maranhão, São Luís, MA, Brasil

**Keywords:** tropical bivalve reproduction, stripping, germinal vesicle breakdown, polar body

## Abstract

The knowledge about the effect of salinity on the physiological mechanism of bivalve reproduction is fundamental to improve production strategies in hatcheries. The present work evaluated the influence of different salinity concentrations (15, 20, 25, 30, 35 and 40 g⋅L^−1^) on pre- and post-fertilization development processes in the clam, *Anomalocardia flexuosa*, oocytes obtained by stripping. Salinity directly interfered with the germinal vesicle breakdown (GVBD) rate and in the cellular stability of unfertilized oocytes. Salinity concentrations between 30 and 35 g⋅L^−1^ provided better percentages of stable GVBD within 120 min, and incubation of oocytes in the salinity range of 30-35 g⋅L^−1^ for a time interval of 80-120 min provided > 80% GVBD. In the post-fertilization analysis, salinity affected the rate of the extrusion of the first and second polar bodies (PB1 and PB2). The release of 50% of the PBs was faster at a salinity of 35 g⋅L^−1^, with an estimated time of 10 min for PB1 and 30 min for PB2. Thus, chromosome manipulation methodologies aiming triploids should be applied at 35 g⋅L^−1^ salinity, with application of post-fertilization shock before 10 min for PB1 retention or before 30 min for PB2 retention.

## Introduction

The production of bivalves in tropical regions has been consolidated as an important market for aquaculture (Nowland et al., 2020; Willer and Aldridge, 2020). However, tropical bivalve larviculture presents a series of obstacles that limits the continuous supply of seeds, essential to aquaculture and restocking (Nowland et al., 2021). One promising species for tropical aquaculture (Lagreze-Squella et al., 2018) is the clam *Anomalocardia flexuosa*, which is distributed from the Caribbean to Brazil (Abbott, 2011), and plays an important role in feeding and in the income of traditional communities (Rios, 2009). Hatchery production methodologies based on feeding, maturation, reproduction, larviculture and settlement events under controlled laboratory conditions have been developed for this species (Lavander et al., 2014; Lagreze-Squella et al., 2018; Lima et al., 2018).

The clam *A. flexuosa,* like most bivalve species, presents external fertilization. Stripping of the gonadal tissue gives access to undeveloped oocytes in maturation stages with intact germinal vesicle structure. This occurs because the oocytes of some species undergo a maturation process during the passage through the oviducts before their release (Colas and Dubé, 1998).

Germinal vesicle breakdown (GVBD) is a natural process occurring in the external environment when metaphase I oocytes are released (Guo et al., 1996). This process is considered as a sign of oocyte maturation. To continue the GVBD stage, the oocytes may go through a period of hydration in the external environment (Melo et al., 2015). The breakdown of the vesicle improves fertilization, decreases polyspermia, and increases efficiency in polyploidy induction processes (Qin et al., 2018), and has been exploited in the hatchery of bivalves (Dégremont et al., 2012).

The process of embryogenesis begins from the time the sperm enters the oocyte and is characterized by the release of two polar bodies, PB1 and PB2 (Colas and Dubé, 1998). Polyploidy can be achieved by the physical or chemical treatment of fertilized eggs to inhibit the exit of PB1 or PB2 (Piferrer et al., 2009).

Environmental factors such as salinity affect the incubation time and the extrusion of polar bodies (Lavander et al., 2014). The knowledge of the effect of environmental variables on oocyte GVBD and the timing of polar bodies release is critical to increase fertilization success in hatcheries, achieve greater control in subsequent embryonic developmental stages, and for adjusting protocols for the use and control of polyploidy (Qin et al., 2018). This study evaluated the influence of salinity on the time of oocyte GVBD and on the extrusion of polar bodies in *A. flexuosa.*

## Methods

### Broodstock

A total of 150 adults were collected in estuarine zone of the state of Maranhão (2º30’03”S, 44º03’40”W) and stored at the State University of Maranhão for the experiments. Approval by the Ethics Council for the Use of Experimental Animals does not apply to the invertebrate group, in accordance with the Brazilian law 11.794/08 (Brasil, 2008).

Clams were cleaned with a sodium hypochlorite solution (2%) and then stored in a 100 L tank in water treated by cartridge filters (50, 25, and 5 μm), biological filter (containing different media and nitrifying bacteria), and UV. The organisms were maintained at a salinity of 30 g⋅L^−1^, temperature of 24 °C, and constant aeration for 4 h of depuration in a Recirculating Aquaculture System (RAS). After this period, the animals were left out of the water overnight at 24 °C to prevent the release of gametes into the water.

### Gametes

For sex identification, each individual was carefully opened using a knife and the gonadal tissue was observed under an optical microscope. Gametes from 30 females were obtained by stripping using a surgical blade. Subsequently, they were mixed and filtered in a 100 µm mesh and rinsed in 25 μm. A dilution of the gametes was carried out to reach a final rate of 50 oocytes/mL in each experimental unit.

### GVBD at different salinities

The effect of salinity on GVBD was evaluated at salinities of 15, 20, 25, 30, 35, and 40 g⋅L^−1^, with five replicates per treatment. The marine water used in the experiment was collected at sea (original concentration of 42 g.L^−1^), filtered and sterilized in a UV filter in the laboratory. The dilution for the different saline concentrations was carried out by adding autoclaved freshwater.

**Figure 1:**
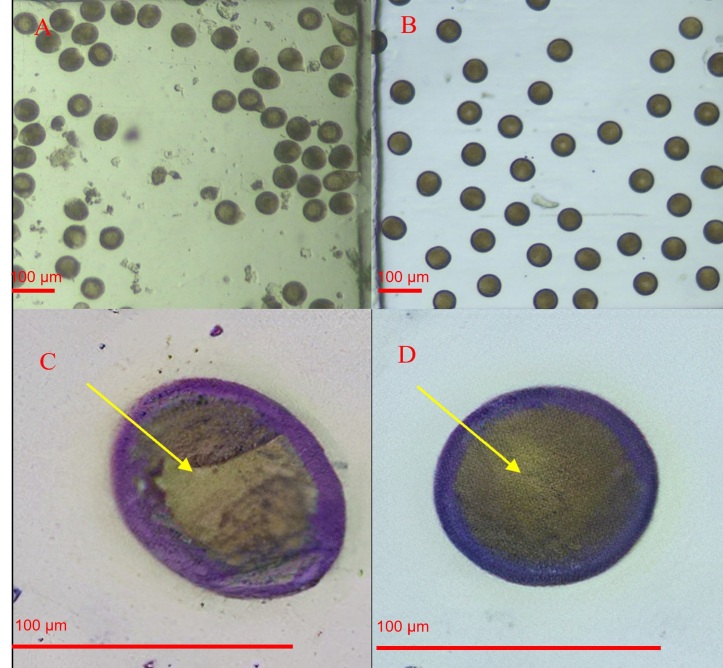
Images registered in the program Mosaic V.2.2.1 with a camera attached to the microscope showing the appearance of *Anomalocardia flexuosa* oocytes before and after germinal vesicle breakdown (GVBD); **A**: Image of a set of oocytes without uniformity soon after undergoing the stripping process observed under 4X lens; **B**: Set of oocytes after hydration showing the GVBD under 4X lens; **C**: Image of a magnified oocyte before hydration with an arrow in yellow highlighting the structure of the intact germinal vesicle observed under 10X lens; **D**: Image of an oocyte magnified after hydration and with a yellow arrow indicating the uniformity of the nucleus as a function of the GVBD observed at 10X lens.

The experimental units were thermostat regulated at 26 °C, which was considered as the ideal temperature for meiosis of the species (Lavander et al., 2017). Diluted oocytes were distributed in 100 mL beakers, from which 1 mL aliquot was removed with the aid of a pipette for observation of the progression of GVBD (Figure 1). The germinal vesicle breakdown process was observed over a period of 120 min, from the observation of 1ml samples (approximately 50 oocytes) taken every 10 minutes for counting and recording.

### Extrusion of PB1 and PB2 in different salinities

The spermatozoa from 30 males were obtained by stripping, mixed, filtered using 80 µm mesh and quantified by optical microscopy. After 2 h of oocyte hydration for GVBD in different salinities (15, 20, 25, 30, 35, and 40 g⋅L^−1^) and at a constant temperature of 26 °C, the spermatozoa were added to each experimental unit at a ratio of 7 spermatozoa: 1 oocyte, according to the previously described methodology (Melo et al., 2015). The first and second polar bodies (PB1 and PB2) extrusion rates were monitored at 5 min intervals over a period of 60 min using 1 ml samples (approximately 50 oocytes).

The average time to obtain the 50% of PB1 and PB2 extrusions for each treatment were determined as previously recommended (Melo et al., 2015; Lavander et al., 2017).

### Statistical analyses

The averages of the five replicates of each salinity treatment were used to estimate the GVBD and the extrusion of PB1 and PB2 at the different time interval. The trends of the percentages of GVBD and PB released with time at each salinity concentration were estimated by fitting the best likelihood model with a generalized additive model (GAM) and considering a binomial distribution. A two-way analysis of variance (ANOVA) was used to measure treatment effects, and averages were compared using Tukey's post-hoc test at a p ≤ 0.05 significance level.

Statistical analyses were performed in R studio version 4.1.0, and the package “MGCV” (Wood, 2011) was used to run the models. The steps for model selection were performed as previously described (Zuur, 2012; Zuur et al., 2009).

## Results

### Effect of salinity on GVBD

**Figure 2:**
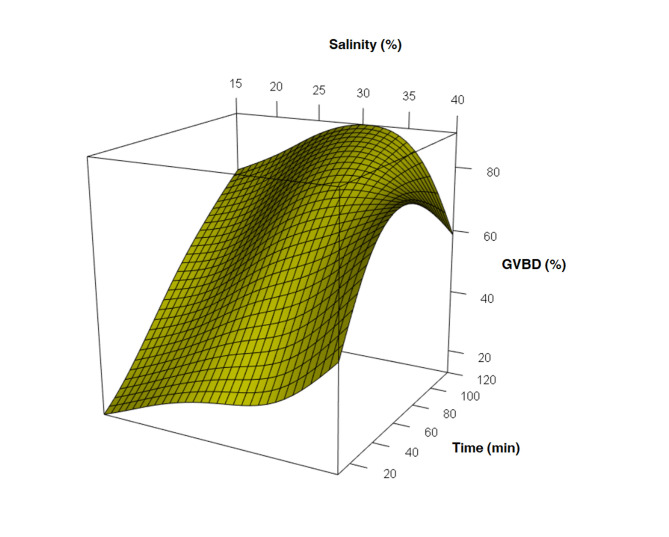
Mean of the percentages of germinal vesicle breakdown of oocytes obtained by stripping from *Anomalocardia flexuosa*.

None of the salinity treatments resulted in 100% GVBD, however, high values were found at 30 and 35 g⋅L^−1^ salt concentrations (93.26 ± 1.04% and 93.76 ± 4.98%, respectively) at 120 min. Also, at 40 g⋅L^−1^ salt concentration 93.99% ± 7 were detected at 50 min, followed by a decrease in the breakdown in subsequent intervals. Salinity treatments at 15, 20 and 25 g⋅L^−1^ presented inferior values <85% after 120 min (Table 1 and Figure 2).

**Table 1:** Germinal vesicle breakdown of *Anomalocardia flexuosa* oocytes under salinity influence.

**Time**	**Percentages of GVBD in the interval from 0 to 120 minutes**					
	**15 g⋅L^–1^**	**20 g⋅L^–1^**	**25 g⋅L^–1^**	**30 g⋅L^–1^**	**35 g⋅L^–1^**	**40 g⋅L^–1^**
T10 (min)	3.24±1.09 ^b^	17.7±1.9 ^ab^	21.84±4.72 ^a^	24.69±10.29 ^a^	17.07±6.33 ^ab^	27.22±12.06 ^a^
T20 (min)	14.95±2 ^c^	25.33±1.32 ^bc^	27.43±7.25 ^bc^	37.16±6.5 ^ab^	31.58±14.65 ^b^	47.63±15.25 ^a^
T30 (min)	22.51±1.5 ^d^	30.36±1.51 ^cd^	34.39±9.73 ^bcd^	48.97±10.94 ^b^	42.72±17.34 ^bc^	73.75±11.55 ^a^
T40 (min)	32.02±4.42 ^c^	35.37±3.97 ^c^	46.8±8.39 ^bc^	51.48±9.89 ^b^	52.52±22.4 ^b^	86.29±12.87 ^a^
T50 (min)	42.62±4.44 ^c^	39.37±1.98 ^c^	53.4±8.6 ^bc^	60.71±8.76 ^b^	64.72±15.24 ^b^	93.99±7 ^a^
T60 (min)	47.39±4.97 ^cd^	42.96±2.56 ^d^	60.17±7.84 ^bc^	69.54±7.86 ^b^	70.79±16.85 ^b^	91.88±5.82 ^a^
T70 (min)	52.12±2.55 ^cd^	44.96±3.41 ^d^	64.31±3.95 ^bc^	74.38±9.14 ^ab^	80.11±15 ^a^	80.08±14.35 ^a^
T80 (min)	54.5±3.96 ^b^	48.6±3.79 ^b^	70.52±2.65 ^a^	82.74±11.44 ^a^	82.66±12.78 ^a^	70.57±10.95 ^a^
T90 (min)	58.24±1.12 ^bc^	55.81±2.13 ^c^	72.22±4.5 ^ab^	87.24±8.89 ^a^	86.96±10.74 ^a^	61.41±6.01 ^bc^
T100(min)	61.6±2.27 ^bc^	61.89±3.19 ^bc^	75.4±4.84 ^ab^	88.39±6.97 ^a^	90.2±8.27 ^a^	55.39±5.79 ^c^
T110(min)	68.84±3.58 ^c^	67.91±3.51 ^c^	78.48±6.72 ^bc^	91.15±3.23 ^ab^	93.76±4.98 ^a^	51.23±7.05 ^d^
T120 (min)	73.75±1.86 ^b^	75.12±2.74 ^b^	82.4±7.06 ^ab^	93.26±1.04 ^a^	93.76±4.98 ^a^	44.8±6.13 ^c^

The mean number of oocytes analyzed per time interval was 50. Different superscript letters in each indicator indicate statistical differences (p < 0.05).

Most of the treatments showed an increase in the percentage of GVBD as salinity raised, but in the 40 g⋅L^−1^ salinity treatment, the oocytes started to break up after 60 min to generate a significant loss until 120 min.

### Effect of salinity on the extrusion of PB1 and PB2

**Figure 3:**
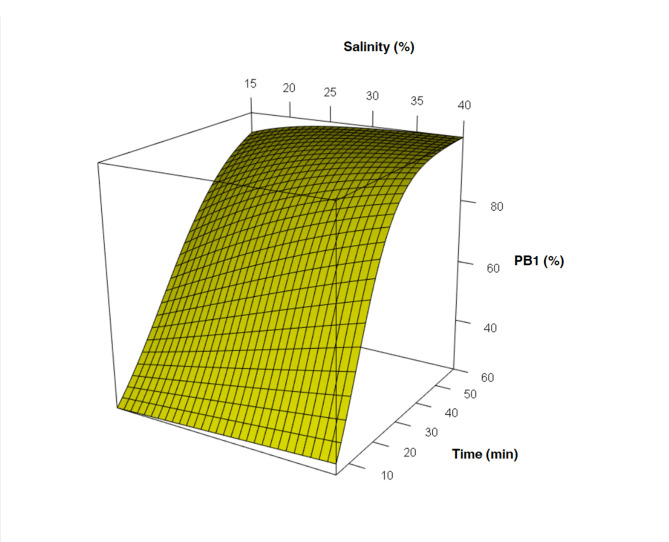
Mean of the percentages of polar body 1 release from fertilized eggs of *Anomalocardia flexuosa*.

Fertilization was conducted right after 120 min to guarantee maximum level of GVBD considering the previous section results. The extrusion of 50% PB in 20, 25, 30, and 40 g⋅L^−1^ treatment groups occurred nearly within the first 15 min. For the 15 g⋅L^−1^, the average time was 25 min, while for 35 g⋅L treatment group it happened in the first 10 min (Table 2 and Figure 3).

**Table 2:** Percentages of polar body 1 (PB1) extrusion of *Anomalocardia flexuosa* at each 5 min interval.

**Time**	**Time to reach 50% of PB1 release**					
	**15 g⋅L^–1^**	**20 g⋅L^–1^**	**25 g⋅L^–1^**	**30 g⋅L^–1^**	**35 g⋅L^–1^**	**40 g⋅L^–1^**
T10 (min)	28.06±3.18 ^c^	29.24±2.23 ^c^	39.44±1.13 ^b^	34.75±4.83 ^bc^	52.22±5.05 ^a^	40.67±7.73 ^b^
T15 (min)	37.68±5.13 ^c^	50.77±1.97 ^b^	46.39±2.29 ^bc^	48.89±3.06 ^b^	74.57±4.61 ^a^	52.28±8.22 ^b^
T20 (min)	43.69±3.01 ^d^	66.83±6.16 ^b^	53.87±3.26 ^c^	58.45±4.89 ^bc^	78.2±3.96 ^a^	63.13±4.56 ^b^
T25 (min)	51.73±0.9 ^e^	78.58±1.99 ^ab^	60.83±1.37 ^de^	63.4±3.2 ^cd^	87.12±5.6 ^a^	71.78±6.56 ^bc^

The mean number of oocytes analyzed per time interval was 50. Different superscript letters indicate statistical differences (p < 0.05).

**Figure 4:**
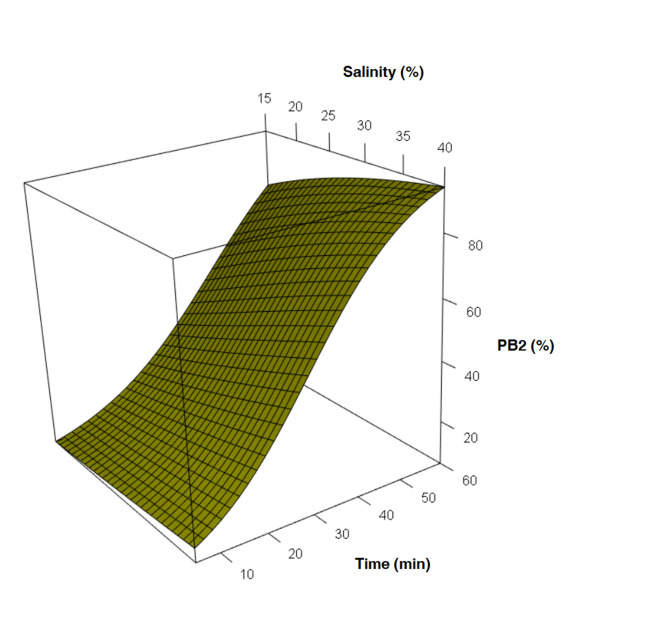
Mean percentage of polar body 2 release from fertilized eggs of *Anomalocardia flexuosa*.

For the extrusion of PB2, in treatments at 20, 25, and 30 g⋅L^−1^, nearly 50% was achieved in the first 35 min, while that rate was accomplished in the first 30 min for 35 and 40 g⋅L^−1^ s (Table 3 and Figure 4). Yet for 15 g⋅L^−1^, extrusion was accomplished later, in 45 min.

**Table 3:** Percentages of polar body 2 (PB2) extrusion of *Anomalocardia flexuosa* at each 5 min interval.

**Time**	**Time to reach 50% of PB2 release**					
	**15 g⋅L^–1^**	**20 g⋅L^–1^**	**25 g⋅L^–1^**	**30 g⋅L^–1^**	**35 g⋅L^–1^**	**40 g⋅L^–1^**
T30 (min)	21.51±5.32 ^c^	39.28±0.65 ^ab^	41.32±0.96 ^ab^	35.16±4.23 ^bc^	50.48±11.94 ^a^	45.39±4.43 ^ab^
T35 (min)	25.43±9.17 ^c^	46.48±6.37 ^ab^	44.91±2.25 ^b^	47.26±7.31 ^ab^	60.38±6.48 ^a^	58.7±0.99 ^ab^
T40 (min)	33.08±9.37 ^c^	56.59±5.76 ^ab^	47.44±1.29 ^b^	55.58±6.27 ^ab^	68.72±10.66 ^a^	67.31±2.07 ^a^
T45 (min)	45.58±6.23 ^d^	62.85±6.71 ^ab^	56.66±3.92 ^cd^	60.17±6.42 ^c^	78.7±9.45 ^a^	75.07±3.92 ^ab^

The mean number of oocytes analyzed per time interval was 50. Different superscript letters indicate statistical differences (p < 0.05).

## Discussion

### Effect of salinity on GVBD

Sodium-potassium antagonism is largely responsible for the oocyte germinal vesicle maintenance (Allen, 1953). The presence of Ca^2+^ ions, on the other hand, is essential for triggering vesicle degradation (Colas and Dubé, 1998). Thus, the salt concentration during hydration of oocytes obtained by stripping directly influences the GVBD (Qin et al., 2018). According to Allen and Bushek (1992), the “immersion time” in seawater is important for synchronization of oocytes that are in prophase I. Thus, GVBD is a signal that meiosis is preparing to advance to the metaphase I stage until fertilization (Eudeline et al., 2000).

The present study suggests the existence of an association between increasing salinity concentrations and GVBD rates. The obtained results suggested that there are important aspects of biochemical and biophysical characteristics of *A. flexuosa* oocytes, such as plasma membrane permeability and osmotic tolerance that interfere on GVBD rates. In hypertonic medium, the cells first react by shrinking owing to the exit of water from the cellular structure. This phenomenon is followed by cell swelling following the entry of water to maintain the osmotic balance (Salinas-Flores et al., 2008).

*A. flexuosa* is characterized as a species tolerant to variation in high salinities (Lagreze-Squella et al., 2018) and, therefore, can adapt its reproduction to the environmental conditions. Based on our results, however, the oocyte stability seems to be associated with a tolerance limit for high saline concentrations. The osmotic tolerance limit may be exceeded if cells undergo excessive shrinkage or swelling that may lead to irreversible injury (Salinas-Flores et al., 2008). Although the salinity of 40 g⋅L^−1^ had a higher catalytic ability for GVBD in our study, the effort to maintain the osmotic balance seemed to be critical for oocytes, leading to the rupture of the cell structure after 60 min.

Different studies have already pointed out that there are factors that can disturb embryogenesis and larval development such as gonadal maturation, oocyte maturation, and polyspermia in bivalves (Lavander et al., 2011; Qin et al., 2018). The larviculture of *A. flexuosa* is characterized as a stage strongly affected by factors such as temperature and salinity, water quality, feeding, and management (Oliveira et al., 2016). In this sense, identifying the best conditions for fertilization can be a tool to help seed production in hatchery. For *A. flexuosa*, 30 and 35 g⋅L^−1^ salinity in the time between 80 and 120 min were the optimum range of hydration with higher percentages of GVBD.

### Effect of salinity on the 50% release of PB1 and PB2

The use of polyploidy has become popular in bivalve aquaculture (Ma et al., 2019), either due to gonadal sterility (Piferrer et al., 2009; Dheilly et al., 2014; Zhang et al., 2017), to the increase in the cell volume and lack of compensation of cell number (Guo and Allen, 1994), or even to the increased heterozygosity that promote larger and faster growth in bivalves (Guo et al., 2009; Yang and Guo, 2018).

There are different alternatives to obtain triploid bivalve individuals. One of them refers to the possibility of interrupting the meiotic process by inhibiting the extrusion of PB1 or PB2 (Piferrer et al., 2009). For this purpose, the determination of the moment of the exit of 50% of PB1 or PB2 after fertilization is important as a reference for the application of inducers (Guo et al., 1996; Lavander et al., 2017; Melo et al., 2015). As in GVBD, salinity can also impact post-fertilization processes such as the timing of release or inhibition of polar bodies in bivalve mollusks (Ma et al., 2019).

In the present study, we were unable to detect an explicit association between increasing salinity and the speed of events, as observed in GVBD evaluation. However, there was an optimal range for the extrusion of both polar bodies that occurred from 20 to 35 g⋅L^−1^ salinity at an interval of 10 to 20 min for PB1 and 30 to 40 min for PB2. The time to initiation of post-fertilization treatment and its duration are the main aspects influencing the success rate of polyploidy and embryo survival (Allen and Bushek, 1992; Eudeline et al., 2000).

Salinities of 15 g⋅L^−1^ may hold back the induction to triploidy because the longer the process time for the release of PB1 or PB2 during chromosome manipulation, the higher are the chances of anomalies occurrence (Qin et al., 2018). Although 40 g⋅L^−1^ concentration showed high percentages of extrusion of PB1 and PB2, the anticipated cell lysis process before fertilization made this treatment unfeasible. A previous report (Guimarães et al., 2008) showed that salinity above 40 g⋅L^−1^ can be lethal in the early life of estuarine bivalves.

Another study with *A. flexuosa* (Lavander et al., 2017) also observed that the highest salinity tested (35 g⋅L^−1^) resulted in better extrusion rates of PB1 and PB2 as compared to 15 and 25 g⋅L^−1^, corroborating the hypothesis that salinity interferes with the exit time of polar bodies. On the other hand, under the same conditions of temperature and salinity (26°C and 35 g⋅L^−1^), these authors found higher extrusion rates, such as of 70% for PB1 until the first 10 min and 62.67% for PB2 after 16 min of fertilization. The discrepancy in the results may be attributed to the methodological differences of observation, prediction analysis, and mainly the methodology of obtaining gametes. Using stripping instead of natural spawning to obtain gametes configures a more controlled strategy for the various treatments applied. However, oocytes obtained by stripping will be in different stages of maturation (Downing and Allen, 1987), contrasting to those obtained by natural or induced release (Lavander et al., 2017).

Regardless of the method applied for spawning, either natural or stripping, the induction of triploidy is most suitable at 35 g⋅L^−1^ salinity, with the possibility of carrying post-fertilization shocks before 10 min for PB1 retention or before 30 min for PB2 retention. According to a previous study (Yang and Guo, 2018), the formation of triploid clams by retention of both PB1 and PB2 is feasible.

Unlike bivalves from temperate climate where temperature largely influences spawning (Camacho et al., 2011; O’Connor et al., 2008), those from tropical regions are more influenced by salinity in the reproductive cycle (Nowland et al., 2021) because temperature does not change much throughout the year (Paixão et al., 2013). A previous study (Lavander et al., 2011) identified that *A. flexuosa* species presented the fullest gonads during the dry period with salinity > 35 g⋅L^−1^ and released gametes during the rainy period with salinities between 25 and 35 g⋅L^−1^. Thus, the species may present a synchronous behavior with the rainy period.

The information that spawning occurs more frequently in monsoon and at intermediate salinities (Lavander et al., 2011) along with the results of the present work that there is an optimal range for GVBD and post-fertilization processes corroborate the idea that there is a reproductive strategy of this animal that is intrinsically related to salinity.

It is also necessary to consider the influence of genetic factors of the population under analysis. Different geographic populations of the same species accumulate alleles that reflect environmental adaptability (Kim et al., 2014; Ren et al., 2016). Organisms with external fertilization can develop their gametic plasticity to adapt to environmental conditions (Lettieri et al., 2019). However, the results presented herein provide important information for the control of the reproductive process and on chromosome manipulation, suggesting that for achieving triploidy in animals maintained at 35 g⋅L^−1^ salinity, the application of post-fertilization shocks should be carried out before 10 min for PB1 or before 30 min for PB2 retentions, respectively.

## Conclusion

We concluded that in the tropical clam (*Anomalocardia flexuosa*) salinity plays a key role on the pre- and post-fertilization stages with best ranges between 30 and 35 g⋅L^−1^ for GVBD and between 20 to 35 g⋅L^−1^ for PB1 and PB2 extrusions. These findings have implications not only for chromosome manipulation approaches aiming triploidy as well as for the reproductive management in hatcheries.
